# Another step toward DNA selective targeting: Ni^II^ and Cu^II^ complexes of a Schiff base ligand able to bind gene promoter G-quadruplexes†
†Electronic supplementary information (ESI) available. CCDC 1451694–1451696. For ESI and crystallographic data in CIF or other electronic format see DOI: 10.1039/c6dt00648e



**DOI:** 10.1039/c6dt00648e

**Published:** 2016-04-04

**Authors:** Alessio Terenzi, Daniela Lötsch, Sushilla van Schoonhoven, Alexander Roller, Christian R. Kowol, Walter Berger, Bernhard K. Keppler, Giampaolo Barone

**Affiliations:** a Institute of Inorganic Chemistry , University of Vienna , Waehringerstr. 42 , A-1090 Vienna , Austria . Email: alessio.terenzi@univie.ac.at; b Research Platform “Translational Cancer Therapy Research” , University of Vienna and Medical University of Vienna , Vienna , Austria; c Department of Medicine I , Institute of Cancer Research and Comprehensive Cancer Center , Medical University Vienna , Borschkegasse 8a , A-1090 Vienna , Austria; d Dipartimento di Scienze e Tecnologie Biologiche , Chimiche e Farmaceutiche , Viale delle Scienze , Edificio 17 , 90128 Palermo , Italy . Email: giampaolo.barone@unipa.it

## Abstract

DNA G-rich sequences are able to form four-stranded structures organized in stacked guanine tetrads. These structures, called G-quadruplexes, were found to have an important role in the regulation of oncogenes expression and became, for such a reason, appealing targets for anticancer drugs. Aiming at finding selective G-quadruplex binders, we have designed, synthesized and characterized a new water soluble Salen-like Schiff base ligand and its Ni^II^ and Cu^II^ metal complexes. UV-Vis, circular dichroism and FRET measurements indicated that the nickel complex can stabilize oncogene promoter G-quadruplexes with high selectivity, presenting no interactions with duplex DNA at all. The same compound exhibited dose-dependent cytotoxic activity in MCF-7 breast cancer cells when combined with lipofectamine as lipophilic carrier.

## Abstract 2

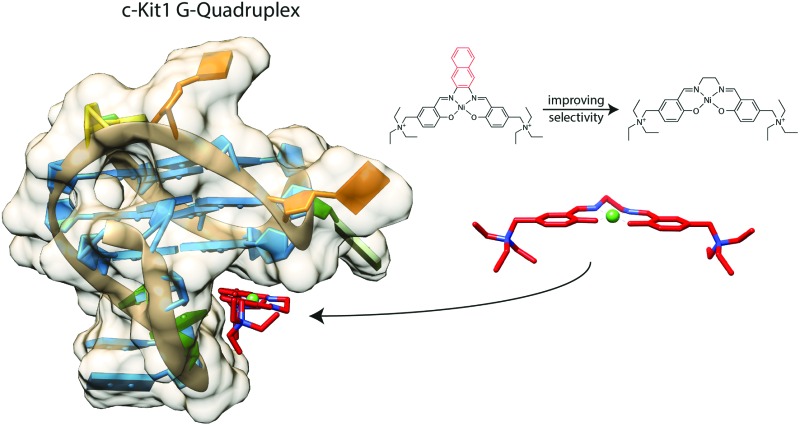
Water-soluble Schiff base Ni^II^ complex in the binding pocket of the oncogene G-quadruplex *c-Kit1*.

## Introduction

DNA can adopt a variety of conformations based on particular sequence motifs that are different from the double-helical B-DNA (ds-DNA) secondary structure. These non-B-DNA structures have attracted an exponentially growing interest as key targets for novel anticancer drugs, due to their involvement in cellular carcinogenic pathways.^1^ Of particular appeal are G-quadruplexes (G4s) which are G-rich sequences capable of forming 4-stranded structures organized in stacked guanine tetrads connected by looping bases. Such structures are not randomly located within the human genome but are overrepresented in telomeres, where they inhibit the activity of telomerase, and in certain gene promoters, *e.g.* oncogenes, with important functions in transcriptional regulation.^2^

In the last 15 years several small molecules, including metal complexes,^3^ able to bind G4 structures have been reported.^4–6^ Due to the heterogeneity of G4 conformations, the selective targeting of these structures with small molecules is a promising anticancer strategy, especially *via* specific down-regulation of oncogene expression.^1^ Nevertheless, the development of novel G4 binders able to reach reasonable high selectivity for specific G4s in oncogene promoters, together with an excellent target affinity (*K*_b_ in the nanomolar range), was rarely achieved.^7^

In the last few years, Vilar,^8,9^ Thomas,^10^ Ralph^11^ and co-workers reported on series of Salen-like and Salphen-like metal complexes with excellent quadruplex DNA binding properties. At the same time, our group developed Ni^II^, Cu^II^ and Zn^II^ Schiff base Salphen-like complexes with DNA-binding ability and, in some cases, good G4 over ds-DNA selectivity.^12–15^

**Fig. 1:**
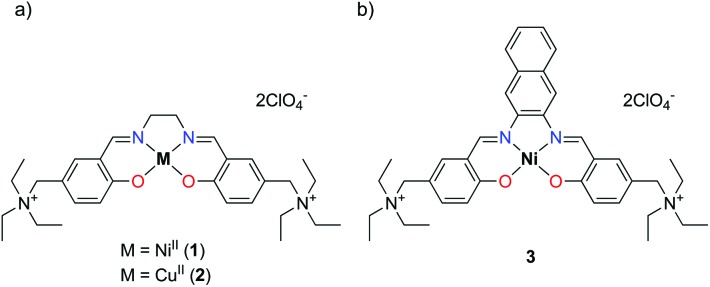
Structures of (a) the newly synthesized Ni^II^ (**1**) and Cu^II^ (**2**) metal complexes and of (b) the Salnaphen-like Ni^II^ complex (**3**).

With the aim of improving the G4 selectivity of our compounds, hence avoiding ds-DNA intercalation and promoting discriminatory binding to peculiar G4 structures, we present in this study the synthesis and characterisation of a novel Salen-like ligand and its nickel(ii) and copper(ii) complexes, **1** and **2** (Fig. 1a). Moreover, we investigated their affinity toward a number of G4 motifs, their biological evaluation toward human cancer cell lines and a molecular model corroborating the experimental results. In addition, we compare the results for this novel compounds with those recently obtained for the Ni^II^ Schiff base complex **3** (Fig. 1b), with a naphthalene moiety in the N,N′ bridge, possessing remarkable anticancer properties and the ability to selectively bind G4s structures over ds-DNA.^14,15^

## Results and discussion

### Synthesis and characterisation

**Scheme 1:**
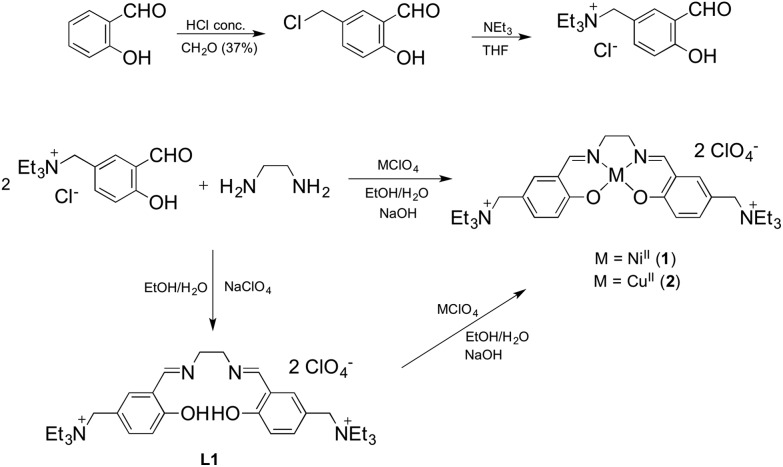
Reaction pathway for the synthesis of compounds **1**, **2** and the ligand **L1**.

The Ni^II^ and Cu^II^ complexes **1** and **2** were prepared in a one step synthesis involving, simultaneously: (i) the condensation of the salicylaldehyde and the diamine to form the Schiff base ligand, (ii) the deprotonation of the slightly acidic OH group by a strong base (NaOH) and (iii) the coordination of the metal centre by adding the corresponding perchlorate salt. The two-step synthetic procedure, with the isolation of the ligand **L1** and its further complexation, was also performed with analogous results (Scheme 1).

**Fig. 2:**
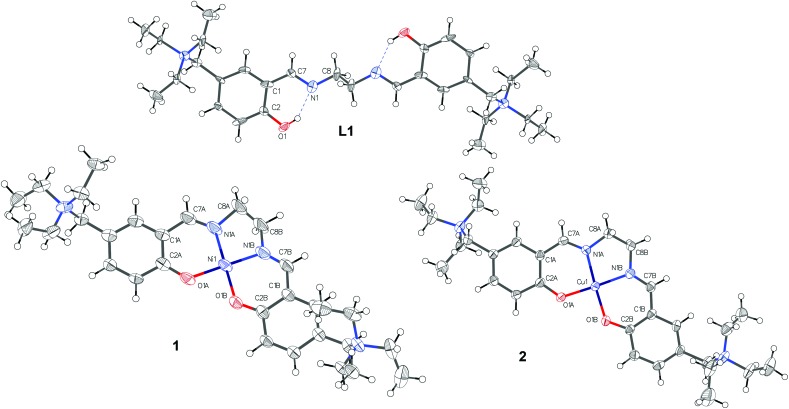
Molecular Structures of **L1**, **1** and **2** drawn with 50% displacement ellipsoids. CDCC codes are ; 1451696, ; 1451694 and ; 1451695 for **L1**, **1** and **2**, respectively.

Crystals suitable for X-ray data collection were obtained by slow evaporation of a MeOH solution of **L1** and by diffusion of diethyl ether into a MeOH solution of **1** and **2** at room temperature. The results of the X-ray diffraction studies are shown in Fig. 2. The single bond between C8 and the symmetric equivalent C8′ (2 – *x*, –*y*, 1 – *z*) of **L1** is arranged as “anti” conformer (exactly 180°, because of symmetry reasons), with N1 and N1′ of the ehylenediamine in a trans configuration. Complexation of the ligand **L1** with Ni^II^ and Cu^II^ led to metal complexes with slightly different geometries. They display molecular configurations and geometries that fall within the typical ranges for these types of compounds.^13^ In detail, due to their coordination bonds, Cu^II^ and Ni^II^ atoms force the distorted ligand **L1** in a square planar disposition with a N1/N1′ *cis* configuration. As expected, the nickel complex **1** is perfectly planar while the copper complex has a distorted geometry. Accordingly, the torsion values and out of plane shifts of the atoms of the metal coordination sphere are presented in Tables S9 and S10,† and are represented in Fig. S4.†

### DNA-binding

#### FRET

FRET (Fluorescence or Förster Resonance Energy Transfer) melting assays^16^ were performed to test the selectivity of our compounds using one double stranded DNA model oligonucleotide (ds-DNA) and six different quadruplex sequences (see Table 1). Stabilization of a folded DNA conformation corresponds to an increase in its melting temperature. In detail, we used the human telomeric *h-Telo*,^17,18^ and four G4s from gene promoters: *c-Myc*,^19^ *c-Kit1*,^20^ *bcl2*,^21^ and *h-TERT*.^22^

**Table 1:** 5′-3′ sequences of the oligonucleotides used for FRET measurements together with their G4 conformation type in KCl buffered solutions. In bold the 4 runs of guanines responsible of the quadruplex formation are shown

Oligos	Sequence	Strands orientation
*h-Telo*	A**GG G**TT A**GG G**TT A**GG G**TT A**GG G**	Mixed parallel–antip.
*c-Myc*	TGG GGA **GGG** T**GG G**GA **GGG** T**GG G**GA AGG	Parallel
*c-Kit1*	A**GG G**A**G GG**C **G**CT **GGG** AGG AG**G G**	Parallel
*h-TERT*	G**GG G**GC T**GG G**CC GGG GAC CCG GGA GGG GTC GGG ACG **GGG** C**GG G**G	Mixed parallel–antip.
*bcl2*	AGG GGC **GGG** CGC **GGG** AGG AAG G**GG G**C**G GG**A GCG GGG CTG	Mixed parallel–antip.
*TERRA*	UUA **GGG** UUA **GGG** UUA **GGG** UUA **GGG**	Parallel
ds-DNA	TATAGCTATA-Heg-TATAGCTATA	Double helical

The exact sequences and their folding G4 topology in KCl buffered solution are listed in Table 1. All of them are conjugated with the fluorophore FAM at the 5′ extremity and with the quencher TAMRA at the 3′ extremity in order to be used for FRET experiments. FAM/TAMRA conjugated TATAGCTATA-Heg-TATAGCTATA sequence was used as a oligonucleotide model able to self arrange in double-helical conformation in physiological conditions.

5,10,15,20-Tetrakis(1-methyl-4-pyridinio)porphyrin (TMPyP), which displays high affinity for both duplex and G4 DNA, regardless of its conformation,^23,24^ and **3**, which displays preference for G4 structures over ds-DNA but essentially no selectivity among quadruplexes,^14^ were used as controls.

**Fig. 3:**
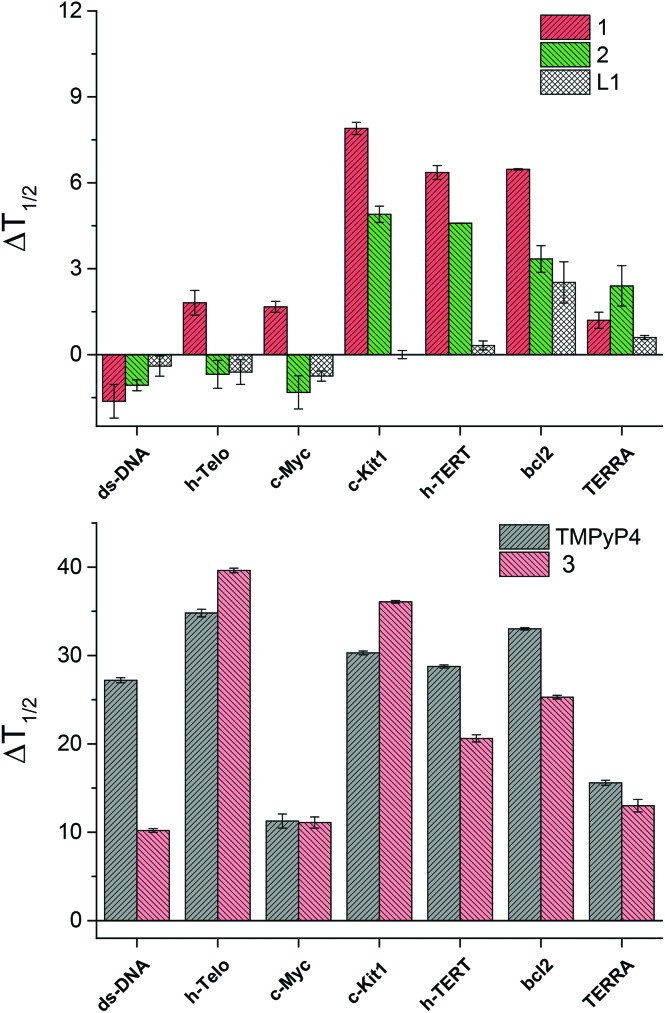
FRET stabilization ability of compounds **1**, **2** and **L1** (top) and of the control binders, TMPyP4 and **3** (bottom), upon binding to the selected oligonucleotides. Buffer: 60 mM potassium cacodylate, pH 7.4.

For each experiment, the final concentrations of the oligonucleotide and of each binder compound were 0.2 μM and 1 μM, respectively. In Fig. 3 is shown the change in melting temperature (Δ*T*_1/2_) of the selected sequences upon binding of the novel compounds and of the controls.

Strikingly, the **L1** ligand and their metal complexes **1** and **2** do not show any binding affinity for ds-DNA, while compound **1**, and to a lower extent also compound **2**, are able to induce a consistent increase of the melting temperature of *c-Kit1*, *h-TERT* and *bcl2* quadruplexes. In comparison, TMPyP4 and **3** show larger Δ*T*_1/2_ values, hence stronger DNA-binding affinity than the novel compounds, however without strong selectivity between ds-DNA and the G4s.^14^ In addition, TMPyP4 and **3** display no selectivity among the different G4s. Thus, compound **1** shows distinctly improved properties, with 100% selectivity toward G4, compared to ds-DNA, and an additional interesting selectivity among the different G4 conformations.

Moreover, the nickel(ii) complex **1** is a stronger G4 binder than the copper(ii) analogue **2**, emphasizing the impact of the metal centre in their binding ability. X-ray crystallography provides a hint to rationalize this trend, pointing out that the structure of **1** is perfectly planar, while that of **2** is distorted. In fact, this feature could impart the nickel(ii) complex **1** better groove binding properties and, as a consequence, higher Δ*T*_1/2_ values.

**Fig. 4:**
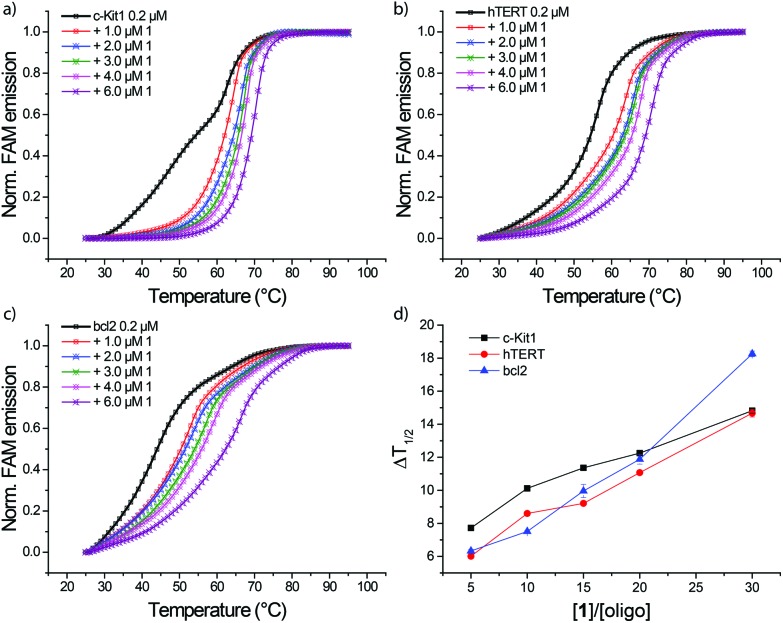
FRET melting profiles of (a) c-Kit1, (b) h-TERT and (c) bcl2 G4 in presence of increasing amounts of **1** in 60 mM potassium cacodylate. (c) Δ*T*_1/2_ trend as function of [**1**]/[oligo] molar ratios.

FRET melting profiles of *c-Kit1*, *h-TERT* and *bcl2*, *vs.* increasing amounts of **1** (Fig. 4), show a concentration dependent stabilization of the three quadruplexes. Interestingly, at lower binder/G4 molar ratios the three sequences show essentially the same Δ*T*_1/2_ trend, with *c-Kit1* slightly more stabilized by **1**, while at higher binder/G4 molar ratios a higher stabilization of *bcl2* quadruplex was observed.

It is worth pointing out that, as already observed for similar nickel(ii) Schiff base complexes, quadruplex stabilization is higher in the presence of aromatic planar groups on the N,N′ bridge.^9–11^ Interestingly, Lecarme *et al.* recently reported on similar nickel(ii)–Salphen complexes with alkyl-imidazolium side-chains that showed high Δ*T*_1/2_ values for telomeric G4 but no-affinity toward ds-DNA.^10^ The novelty of our compound **1** is its selectivity among different G4 conformations, with particular affinity for c-Kit1.

### Circular dichroism and UV-visible studies

**Fig. 5:**
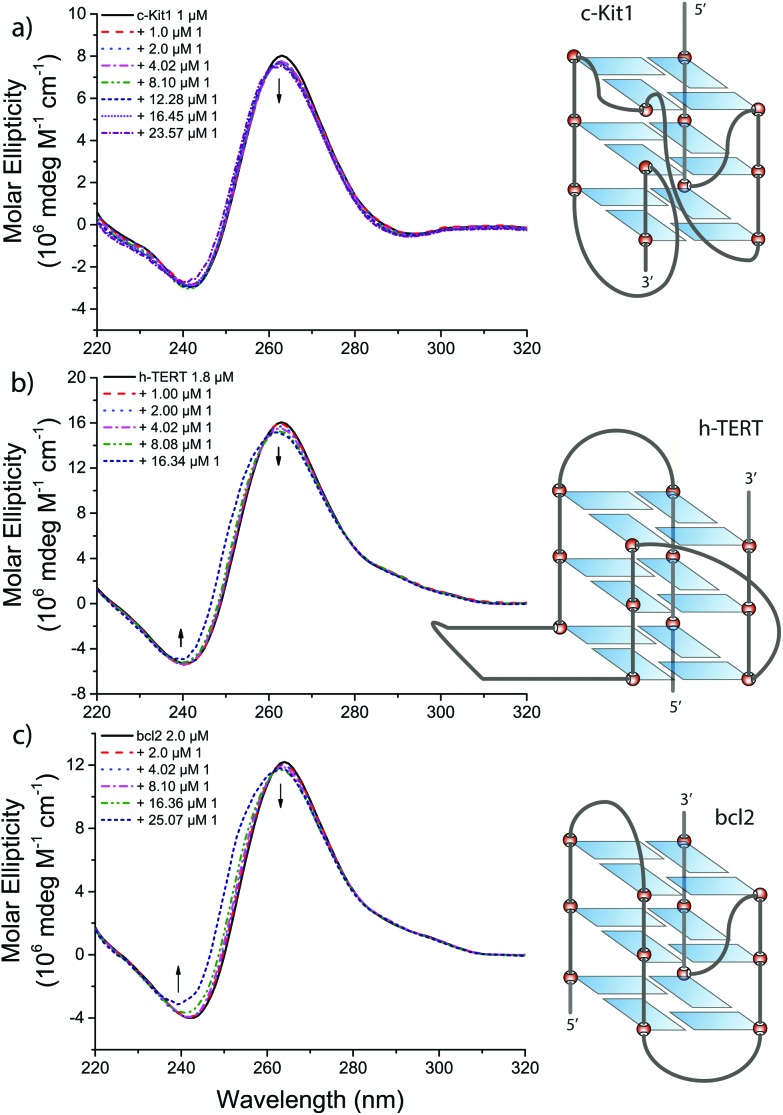
Circular dichroism spectra of (a) c-Kit1, (b) h-TERT and (c) bcl2 G4 in presence of increasing amounts of **1** in 50 mM Tris-HCl and 100 mM KCl.

Intriguingly, the three G4s for which the Ni^II^ compound **1** has higher affinity are known for their peculiar folding patterns (see Fig. 5): (i) the *c-Kit1* sequence, 87 nucleotides upstream of the transcription start site of the human c-Kit gene, forms a G4 with a unique large binding pocket due to discontinuity in the tetrads connection,^20^ (ii) the *h-TERT* sequence forms an atypical quadruplex conformation with two pairs of consecutive G-tracts separated by a 26-base loop arranged in a stable hairpin structure,^22^ and (iii) the *bcl2* promoter, finally, is known to form a mixture of three distinct intramolecular quadruplexes,^25,26^ with the most stable one adopting a novel folding of mixed parallel/antiparallel-stranded structure.^21^

We performed circular dichroism (CD) measurements to monitor the formation of G4 structures and to check whether these structures are preserved upon interaction with our lead compound **1**. Increasing amounts of **1** were added to Tris-HCl/KCl solutions of previously G4-annealed *c-Kit1*, *h-TERT* and *bcl2*, and their CD spectra recorded.

The three sequences produced CD spectra characteristic of G4 structures in a potassium chloride solution (black solid line in Fig. 5a–c). They share a positive maximum at 264 nm and a negative minimum at 240 nm, typical for CD spectra of a parallel G-quadruplex.^27,28^ In addition, *h-TERT* and *bcl2* exhibit a small positive shoulder adsorption in the range 280–300 nm, indicative of a mixed parallel/antiparallel G-quadruplex.^22,25,26^

Upon addition of increasing amounts of **1**, small spectral changes were observed in the CD of the three quadruplexes (Fig. 5), implying that the G4 conformations are preserved after the interaction. Small hypsochromic and hypocromic shifts of the bands around 260 and 240 nm were observed with the most pronounced effects in case of *bcl2* at higher concentration of **1**, in accordance with FRET results.

In order to determine the intrinsic binding constant (*K*_b_) of the **1**-DNA systems, UV-Vis spectra of **1** were recorded in the presence of increasing amounts of duplex and G4 DNA. The characteristic band of compound **1** at 389 nm (black solid line in Fig. S9–S12 ESI†), in a region where DNA is transparent, was affected by the addition of increasing amounts of the selected G4 oligonucleotides, producing hypochromic and bathochromic shifts (see Fig. S10–S12 ESI†), while no effect was observed when ds-DNA was added (Fig. S9, ESI†). DNA-binding constants (see Table 2) were obtained by fitting the absorption data using two equations: one developed by Wolfe *et al.* (reciprocal plot),^29^ recently used for Schiff base metal complexes interacting with G4s,^9^ and the one proposed by Rodger and Nordén (Intrinsic method)^30^ (see ESI† for further details).

**Table 2:** Binding constant values for the **1**-DNAs systems

*K*_b_ (M^–1^)	Reciprocal plot	Intrinsic method
ds-DNA	No interaction	No interaction
*c-Kit1*	(2.4 ± 0.5) × 10^5^	(4.9 ± 0.3) × 10^4^
*h-TERT*	(1.1 ± 0.1) × 10^5^	(3.7 ± 0.4) × 10^4^
*bcl2*	(2.0 ± 0.4) × 10^5^	(3.4 ± 0.3) × 10^4^

Interestingly, data treatment by reciprocal plot method provides *K*_b_ values of roughly one order of magnitude higher. Nevertheless, both approaches corroborate the results obtained by FRET. In fact, no interaction of **1** with double stranded DNA was observed, and a slight preference for *c-Kit1* over the other two G4s was confirmed.

Remarkably, compound **1** demonstrated to be efficiently G4-selective over DNA duplex. In comparison, the control compound **3** shows higher affinity for G4 structures, with *K*_b_ of about 10^6^ M^–1^ for telomeric quadruplex,^14^ but also a high ds-DNA binding constant at *ca.* 10^4^ M^–1^.^14^

### Biological activity

We also evaluated whether the selective G4 stabilizers **1** and **2** exhibit cytotoxic activity. Accordingly, several malignant cell models derived from melanoma (VM01 and VM47), osteosarcoma (U2-OS), glioblastoma (U87MG) and breast cancer (MCF-7) were treated with compounds **1** and **2**. Interestingly, the viability of the investigated cancer cell lines was not affected upon 72 h drug exposure (Fig. S14, ESI†). We hypothesized that the lower lipophilicity of **1** and **2**, compared to **3** (Fig. 1), might inhibit cellular uptake through cell membranes.

**Fig. 6:**
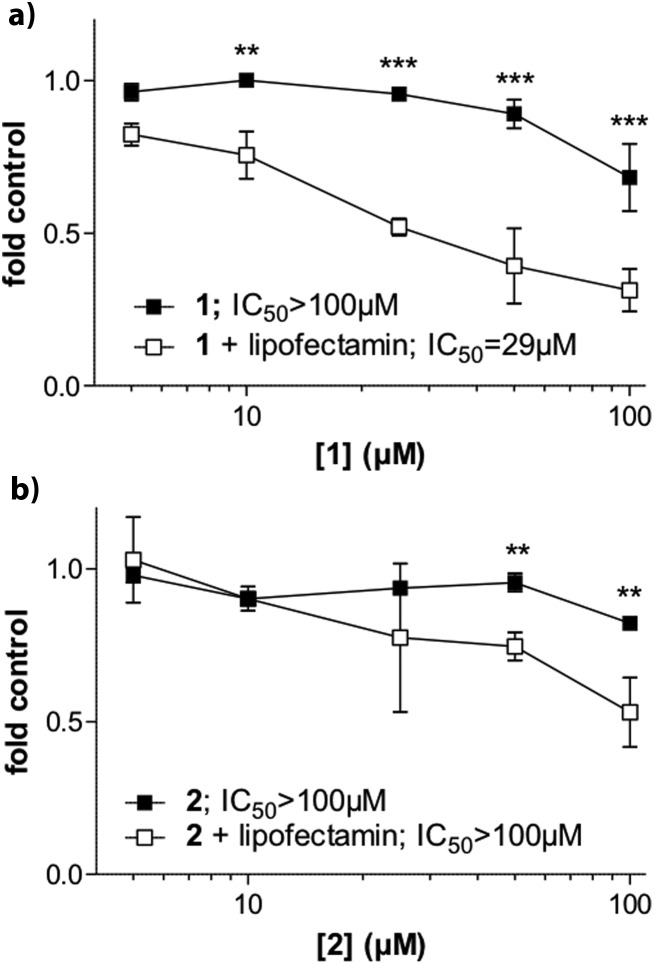
Cytotoxic activity of compound **1** and **2** in MFC-7 breast cancer cells and impact of lipofectamine. Cells were treated with substance **1** (A) and **2** (B) at the indicated concentrations in the presence or absence of lipofectamine. After 72 hours incubation, cell viability was determined by MTT assays. Cytotoxicity levels are additionally expressed as IC_50_ values calculated by Graph Pad prism software using point-to-point function. Statistical analysis was performed by Two-way ANOVA (**P < 0.01; ***P < 0.001).

To test this assumption, we performed cytotoxicity assays in MCF-7 breast cancer cells in the presence of lipofectamine 2000. Lipofectamine is a known lipophilic carrier commonly used as transfection agent *in vitro*,^31^ that we have previously used as transmembrane carrier for copper(ii) and zinc(ii) complexes.^32^ Indeed, compound **1** exhibited increased and dose-dependent cytotoxic activity when combined with lipofectamine (Fig. 6a). The effect was comparable but less pronounced for the copper complex **2** (Fig. 6b).

### Molecular modelling

To get perception of the binding sites of compound **1** within the selected G4 structures, molecular docking simulations were performed (see Computational details). PDB entries ; 2O3M and ; 2F8U were used as 3D models of *c-Kit1* and *bcl2* quadruplexes.

**Fig. 7:**
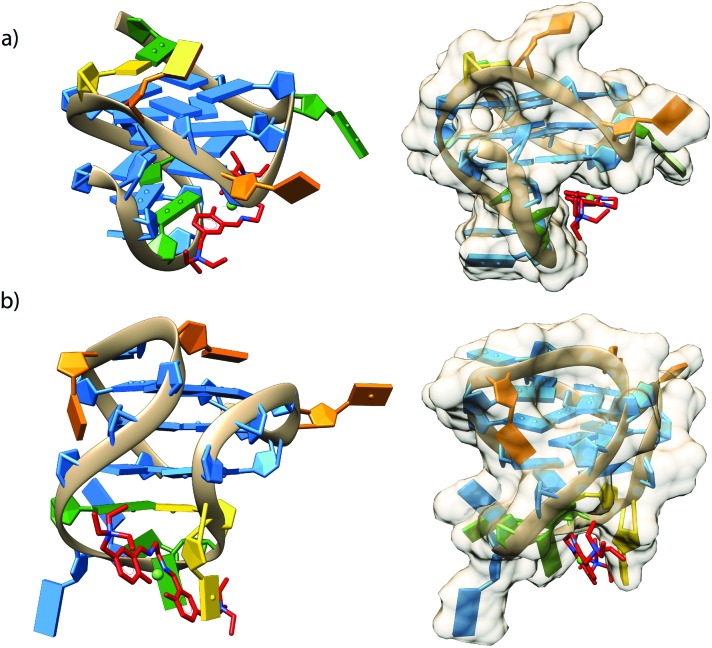
Cartoons showing possible binding sites of **1** with (a) c-Kit1 (PDB entry ; 2O3M) and (b) the bcl2 (PDB entry ; 2F8U) G4 motifs, obtained through the docking study. Images on the left-hand side show side views of **1** (coloured in red) groove-binding; images on the right-hand side show the models from other angles with a van der Waals surface. (Colours: G = blue, C = orange, T = gold, A = green).

Fig. 7 shows the poses with better scores, (see Table 3) extracted and aligned with the G4 3D structures. Docking results revealed that **1** prefers groove-binding over top-stacking for both G4s. Of particular interest is the position of compound **1** within the groove binding pocket of the *c-Kit1* structure. Indeed, it was suggested that this pronounced cleft, not found in any other known G4 so far, may be suitable for selective ligand binding.^1^

**Table 3:** Docking binding scores for compound **1** with c-Kit1 and bcl2 quadruplexes

	Score (kcal mol^–1^)
**1**-*cKit1*	–7.0
**1**-*bcl2*	–5.8
TMPyP-2HRI^*a*^	–7.7

^*a*^Parallel stranded human telomeric quadruplex in complex with the compound TMPyP4 (PDB ID: 2HRI),^33^ is shown as control (pose in the ESI Fig. S15).

The binding features of compound **1**, confirmed *via* both experimental and *in silico* approaches, are distinctly different from known unselective G4 ligands called top/end-stackers, which in fact are usually polyaromatic molecules, such as BRACO-19^34^ and RHPS4,^35^ as well as TMPyP4 and compound **3**,^14^ binding to the ends of the G-tetrads by π-stacking interactions.

To rationalize G4 over DNA-duplex selectivity, from the structural point of view, it can be useful to compare the size of the grooves of double helical DNA and of the considered G4s, obtained from static structural studies: the grooves of *c-Kit1* and *bcl2* G4, where **1** perfectly fits, are about 7 Å (Fig. S16, ESI†), while in double helical B-DNA the minor and major grooves are about 5 Å and 12 Å, respectively.^36–38^ Such data would indicate that the minor and major groove sizes of DNA duplex are too small and too big, respectively, for an efficient non-covalent interaction with **1**.

## Conclusions

We have synthesized and thoroughly characterized a nickel(ii) and a copper(ii) Schiff base Salen-like cationic complexes, **1** and **2**, and spectroscopically studied their binding toward duplex and G-quadruplex DNA in solution. Compared with the reference nickel(ii) compound **3**, bearing a naphtalene group on the N,N′ bridge of the Salen scaffold, the affinity of compounds **1** and **2** slightly decreases toward G-quadruplex DNA, but the G-quadruplex *vs.* duplex DNA selectivity is dramatically enhanced. In fact, both **1** and **2** are not ds-DNA intercalators and, moreover, a preference for the binding to defined G4 sequences is revealed, such as *c-Kit1 vs. h-Telo*.

Furthermore, the planarity of **1**, compared to the distorted geometry of **2**, confirmed by X-ray crystallography, plays an important role in the binding mode, making compound **1** a better G4 binder.

The DNA-binding studies, together with the support of molecular modelling, reveal the occurrence of an unusual “groove binding” mechanism for lead compound **1** with G-quadruplex structures from *c-Kit1*, *bcl2*, and *h-TERT* sequences, different from the well-known top-stacking interaction.

The cationic charge of the complexes and the absence of any aromatic group on the N,N′ bridge, inhibit the crossing of the cell membrane of cancer cell lines. However, in the presence of the lipophilic carrier lipofectamine, both compounds exhibited dose-dependent cytotoxic activity. Interestingly, this was less pronounced for **2** as compared to **1**, in parallel to the G-quadruplex affinity.

Compounds like **1**, recognizing specific groove conformations of G4 structures but being not able to interact with double helical DNA, represent a promising and more selective alternative to G4 top/end-stackers.

## Experimental

### General

Solvents (analytical grade) were all commercial and used without further purification. Chemicals, including 5,10,15,20-tetrakis(1-methyl-4-pyridinio)porphyrin tetra(*p*-toluenesulfonate) (TMPyP), were purchased from Sigma Aldrich or Acros and used as received.

^1^H NMR and ^13^C NMR spectra were recorded at 500.10 MHz and 125.75 MHz, respectively, using a Bruker FT-NMR spectrometer Avance III™ 500 MHz. DMSO-d_6_ was used as solvent for the NMR experiments, with samples concentrations around 2–4 mM. Electrospray ionization mass spectra were recorded on a Bruker Esquire 3000 in positive and negative mode; the samples were dissolved in methanol. Elemental analyses were carried out with a Eurovector EA3000 Elemental Analyzer by the Microanalytical Laboratory of the University of Vienna.

Analysis and plotting of the data were carried out using Origin 9.5 (OriginLab Corp.).

### Synthesis

Compound **3** was synthesized and characterized as recently reported.^14,15^ 5-Chloromethyl salicylaldehyde was synthesised according to literature procedures.^39^ Starting material (5-(triethylammoniummethyl)salicylaldehyde chloride) was prepared from 5-chloromethyl salicylaldehyde and triethylamine in tetrahydrofuran, as previously reported.^14,40^

#### [*N*,*N*′-Bis(5-triethylammoniummethylsalicylidene)-1,2-ethylenediamine](ClO_4_)_2_ (**L1**)

Pure ethylenediamine (27 μl, 0.4 mmol) in MilliQ water (0.5 ml) was added dropwise to 5-(triethylammoniummethyl)salicylaldehyde chloride (220 mg, 0.81 mmol) previously dissolved in H_2_O/EtOH 5 : 1 (5.0 ml). The mixture was stirred at room temperature for 30 min. After the addition of NaClO_4_ (98 mg, 0.80 mmol) a pale yellow precipitate was collected, washed with cold ethanol, diethyl ether and dried under vacuum to give the product (180 mg, yield: 65%).

Crystals suitable for X-ray diffraction analysis were obtained by slow evaporation of a MeOH solution of the compound at room temperature.

^1^H NMR 500 MHz, DMSO, 298 K; *δ* (ppm) 1.30 (t, 18H, *J* = 7.1 Hz, CH_3_); 3.15 (q, 12H, *J* = 7.1 Hz, CH_2_); 3.98 (s, 4H, CH_2_); 4.44 (s, 4H, CH_2_); 6.97 (d, 2H, *J* = 8.6 Hz, Ar–H); 7.46 (dd, 2H, *J* = 8.6, 2.2 Hz, Ar–H); 7.61 (d, 2H, *J* = 2.2 Hz, Ar–H); 8.70 (s, 2H, imminic H); 13.82 (s, 2H, OH).

^13^C{^1^H} NMR, 126 MHz, DMSO, 298 K; *δ* (ppm) 8.0 (CH_3_); 52.1 (CH_2_); 58.6 (CH_2_); 59.4 (CH_2_); 117.8 (Ar, C); 118.1 (Ar, CH); 118.9 (Ar, C); 136.5 (Ar, CH); 137.0 (Ar, CH); 163.1 (Ar, C); 167.1 (imminic CH).

Elemental analysis for C_30_H_48_Cl_2_N_4_O_10_ (**L1**). Found: C, 51.95%, H, 7.05%, N 8.02%; calc.: C, 51.80%, H, 6.96%, N, 8.05%.

#### [(*N*,*N*′-Bis(5-triethylammoniummethylsalicylidene)-1,2-ethylenediiminato)nickel(ii)] (ClO_4_)_2_ (**1**)

5-(Triethylammoniummethyl)salicylaldehyde chloride (135.9 mg, 0.50 mmol) was dissolved in H_2_O/EtOH 5 : 1 (5 ml) and solid NaOH (*ca*. 20.0 mg, 0.50 mmol) was added. To this solution, an aqueous solution of ethylenediamine (16.7 μl in 0.5 ml H_2_O, 0.25 mmol) was added dropwise and the mixture stirred for 30 min. Finally, Ni(ClO_4_)_2_·6H_2_O (91.4 mg, 0.25 mmol) previously dissolved in a minimum amount of water was added dropwise. The resulting mixture was stirred for 6 h at room temperature. The brilliant orange precipitate was filtered and washed with cold water, cold ethanol and diethyl ether, and recrystallized from 50% CH_2_Cl_2_–MeOH solution to afford the compound as an orange solid (yield 106.0 mg, 56%). Crystals suitable for X-ray diffraction analysis were obtained by diffusion of diethyl ether into a MeOH solution of the compound at room temperature.

^1^H NMR 500 MHz, DMSO, 298 K; *δ* (ppm) 1.29 (s, 18H, CH_3_); 3.12 (s, 12H, CH_2_); 3.49 (s, 4H, CH_2_); 4.32 (s, 4H, CH_2_) 6.81 (d, 2H, *J* = 6.9 Hz, Ar–H); 7.26 (d, 2H, *J* = 6.9 Hz, Ar–H); 7.44 (s, 2H, Ar–H); 8.02 (s, 2H, NCH imminic H).

^13^C{^1^H} NMR, 126 MHz, DMSO, 298 K; *δ* (ppm) 7.9 (CH_3_); 51.8 (CH_2_); 58.7 (CH_2_); 59.6 (CH_2_); 113.5 (Ar, C); 120.9 (Ar, CH); 121.0 (Ar, C); 137.4 (Ar, CH); 138.1 (Ar, CH); 163.5 (imminic CH); 165.2 (Ar, C).

Elemental analysis for C_30_H_48_Cl_2_N_4_NiO_11_·H_2_O. Found: C, 46.67%, H, 6.18%, N 6.98%; calc.: C, 46.78%, H, 6.28%, N, 7.27%.

#### [(*N*,*N*′-Bis(5-triethylammoniummethylsalicylidene)-1,2-ethylenediiminato)copper(ii)] (ClO_4_)_2_ (**2**)

5-(Triethylammoniummethyl)salicylaldehyde chloride (135.9 mg, 0.50 mmol) was dissolved in H_2_O/EtOH 5 : 1 (5 ml) and solid NaOH (20.0 mg, 0.50 mmol) was added. To this solution, an aqueous solution of ethylenediamine (16.7 μl in 0.5 ml H_2_O, 0.25 mmol) was added dropwise and the mixture was stirred for 30 min. Finally, Cu(ClO_4_)_2_·6H_2_O (92.6 mg, 0.25 mmol) previously dissolved in a minimum amount of water was added dropwise. The resulting mixture was stirred for 4 h at room temperature. The green precipitate was washed with cold water, cold ethanol and diethyl ether, and recrystallized from hot MeOH to afford the compound as a greenish solid (yield 130.0 mg, 69%). Crystals suitable for X-ray diffraction analysis were obtained by diffusion of diethyl ether into a MeOH solution of the compound at room temperature.

Elemental analysis for C_30_H_48_Cl_2_N_4_CuO_11_·H_2_O. Found: C, 46.24%, H, 6.17%, N 7.01%; calc.: C, 46.48%, H, 6.24%, N, 7.23%.

### X-Ray crystallography

The X-ray intensity data were measured on Bruker D8 Venture (compound **1**) and Bruker X8 Apex2 (compounds **L1** and **2**) diffraktometers equipped with multilayer monochromators, Mo K/a INCOATEC micro focus sealed tube and Kryoflex cooling devices. The structures were solved by direct methods and charge flipping and refined by full-matrix least-squares techniques. Non-hydrogen atoms were refined with anisotropic displacement parameters. Hydrogen atoms were inserted at calculated positions and refined with riding coordinates respectively as rotating groups. The following software was used: frame integration, Bruker SAINT software package,^41^ using a narrow-frame algorithm, absorption correction, TWINABS&SADABS,^42^ structure solution, SHELXS-2013,^43^ OLEX2,^44^ refinement, SHELXL-2013,^43^ OLEX2,^44^ SHELXLE,^45^ molecular diagrams, OLEX2.^44^ Experimental data and CCDC code can be found in Table S1.† Crystal data, data collection parameters, and structure refinement details are given in Tables S2–S8.† Selected bond lengths angles and distances are listed in Tables S9–S12.† Molecular Structures are displayed in Fig. S1–S5.†

### FRET studies

FRET experiment were performed in 96-well plates and run on an Applied Biosystems® 7500 Real-Time PCR cycler equipped with a FAM filter (*λ*_ex_ = 492 nm; *λ*_em_ = 516 nm). All fluoro-labelled oligonucleotides (see Table 1) were purchased from IDT (Integrated DNA Technologies, Belgium) in HPLC purity grade. The FRET probes used were FAM (6-carboxyfluorescein) and TAMRA (6-carboxy-tetramethylrhodamine). As model for ds-DNA the TATAGCTA-Heg-TATAGCTATA sequence was used (Heg linker = [(–CH_2_–CH_2_–O–)_6_]).

The lyophilized strands were firstly diluted in MilliQ water to obtain 100 μM stock solutions. The exact concentration of the oligonucleotide stock solutions was checked measuring the absorbance at 260 nm of the corresponding diluted solutions using the extinction coefficient values provided by the manufacturer.

Stock solutions were diluted to a concentration of 400 nM in 60 mM potassium cacodylate buffer (pH 7.4) and then annealed to form G4 structures by heating to 95 °C for 5 min, followed by slowly cooling to room temperature overnight.

Experiments were carried out in a 96 well plate with a total volume of 30 μl. Final concentration of the oligonucleotides was 200 nM. All compounds, including control TMPyP4, were previously dissolved in DMSO to give 1 mM stock solutions. These were further diluted using 60 mM potassium cacodylate, and added to obtain a final concentration of 1 μM (with a total percentage of DMSO around 0.1%).

The machine was set to perform a stepwise increase of 1 °C every 30 s starting from 25 °C to reach 95 °C, and measurements were acquired after each step. To compare different sets of data, FAM emission was normalised (0 to 1).^16^ *T*_1/2_ is defined as the temperature at which the normalised emission is 0.5. Measurements were made in duplicate or triplicate.

### UV-visible absorption

UV-vis spectra were collected on a PerkinElmer LAMBDA 35 double beam spectrophotometer equipped with a Peltier temperature controller, using 1 cm path-length quartz cuvettes. Lyophilized calf thymus DNA (Sigma-Aldrich) was used a model for ds-DNA. It was resuspended in 1.0 mM tris-hydroxymethyl-aminomethane (Tris-HCl) pH = 7.5 and dialyzed as described in the literature.^46^ DNA concentration, expressed in monomers units ([DNA_phosphate_]), was determined by UV spectrophotometry using 6600 M^–1^ cm^–1^ as molar absorption coefficient at 260 nm.^47^ All experiments were carried in 100 mM KCl, 50 mM Tris-HCl aqueous buffer at pH = 7.5.

The G4 sequences *c-Kit1* (AG_3_AG_3_CGCTG_3_AGGAG_3_), *h-TERT* (G_5_CTG_3_CCG_4_ACCCG_3_AG_4_TCG_3_ACG_4_CG_4_) and *bcl2* (AG_4_CG_3_CGCG_3_AGGAAG_5_CG_3_AGCG_4_CTG), were purchased from IDT (Integrated DNA Technologies, Belgium) in HPLC purity grade. The oligonucleotides were dissolved in MilliQ water to yield a 100 μM stock solution. These were then diluted using 50 mM Tris-HCl/100 mM KCl buffer (pH 7.4) to the desired concentration. The oligonucleotide were folded by first heating the solutions up to 90 °C for 5 min and then by slowly cooling down to room temperature. Concentration of the oligonucleotide solutions was checked measuring their absorbance and using the appropriate extinction coefficients as reported by the manufacturer.

UV-visible absorption spectra were recorded at 25 °C. The titrations were carried out by adding increasing amounts of DNA (ct-DNA or oligos) solution to a metal-complex solution with constant concentration. To ensure that during the titration the concentration of the selected metal complex remained unaltered, for each addition of the DNA solution the same volume of a double-concentrated metal complex solution was added.

### Circular dichroism

Circular dichroism spectra were recorded on a Chirascan™ CD Spectrometer (by AppliedPhotophisics), using 1 cm path-length quartz cuvettes, at 25 °C with the following parameters: range 500–200 nm, bandwidth: 1.0 nm, time per point: 0.5 s, repeats: 4. The titrations were carried out by adding increasing amounts of a metal-complex stock solution to a DNA solution with constant concentration. To ensure that during the titration the concentration of the DNA remained unaltered, for each addition of the complex solution, the same volume of a double-concentrated DNA solution was added. UV-Vis spectra of the same solutions were recorded and are represented in Fig. S6–S8 of the ESI.†

### Biological activity

Malignant cell models derived from melanoma (VM01 and VM47, established at the Medical University Vienna), osteosarcoma (U2-OS, purchased from ATCC, VA, USA), glioblastoma (U87MG, purchased from ATCC) and breast cancer (MCF-7, purchased from ATCC) were used to determine the impact on cell viability. Therefore, 2 × 10^4^ cells per ml were seeded in 96-well plates and allowed to attach for 24 h. Afterwards cells were treated with the compounds at the indicated concentrations in presence or absence of lipofectamine (Thermo Fisher Scientific, Waltham, MA, USA). Upon 72 hour continuous drug exposure, anticancer activity was measured by the 3-(4,5-dimethylthiazol-2-yl)-2,5-diphenyltetrazolium bromide (MTT)-based vitality assay (EZ4U; Biomedica, Vienna, Austria) following the manufacturer's instructions.^48^ Cell viability was determined using the Graph Pad Prism software (version 5; GraphPad Software, San Diego, CA) and response to the compounds was expressed as IC50 values (drug concentrations inducing a 50% reduction of cell number in comparison to untreated controls).

### Computational details

Molecular docking was performed by the software AutoDock Vina 1.1.2,^49^ using default parameters. The Protein Data Bank files PDB ID: ; 2O3M and PDB ID: ; 2F8U were used as models of *c-Kit1* and *bcl2* G-quadruplex receptors, respectively. The crystallographic geometry of compound **1**, to be used in the docking studies, was previously fully optimized by DFT calculations implemented in the Gaussian09 program package,^50^ using the B3LYP functional,^51–53^ the Lanl2dz pseudopotential basis set for nickel,^54^ and the 6-31G(d,p) basis set for the other atoms.^55,56^

The Autodock Tools 1.5.6 software was used to add and merge non-polar hydrogens to the receptors and to assign the rotatable bonds to the ligands.^57^ A grid box large enough to allow any possible ligand–receptor complex (blind-docking) was created. In particular, grid size for ; 2O3M was set to 30 Å × 30 Å × 26 Å points with grid spacing of 1.0 Å and a grid center of –1.033, 2.235 and –1.266. Grid size for ; 2F8U was set to 26 Å × 26 Å × 26 Å points with grid spacing of 1.0 Å and a grid center of 0.0, 0.0 and 0.0. Validation of AutoDock Vina was performed running a docking calculation on a parallel stranded human telomeric quadruplex in complex with the compound TMPyP4 (PDB ID: ; 2HRI),^33^ confirming both the position of the ligand and the reasonability of the scoring values (see ESI, Fig. S14†). Figures were rendered using Chimera software.^58^
