# Supplementary material for: Another step toward DNA selective targeting: NiII and CuII complexes of a Schiff base ligand able to bind gene promoter G-quadruplexes
Source: Dalton Trans. 2016 Apr 4;45(18):7758–67. doi: 10.1039/c6dt00648e (PMC5048337; doi:10.1039/c6dt00648e)
Supplement: Supplementary file 1 [file DT-045-C6DT00648E-s001.pdf]

Electronic Supplementary Material (ESI) for Dalton Transactions.
This journal is © The Royal Society of Chemistry 2016

Another Step Toward DNA Selective Targeting: Ni^{II} and Cu^{II} Complexes of a Schiff Base Ligand able to bind gene promoter G-Quadruplexes

Alessio Terenzi,[a,b] Daniela Lötsch,[c] Sushilla van Schoonhoven,[c] Alexander Roller,[a] Christian R. Kowol,[a,b] Walter Berger,[b,c] Bernhard K. Keppler,[a,b] Giampaolo Barone.*[d]*

[a]*Institute of Inorganic Chemistry, University of Vienna, Waehringerstr. 42, A-1090 Vienna, Austria.*

[b]*Research Platform "Translational Cancer Therapy Research", University of Vienna and Medical University of Vienna, Vienna, Austria.*

[c]*Department of Medicine I, Institute of Cancer Research and Comprehensive Cancer Center, Medical University Vienna, Borschkegasse 8a, A-1090 Vienna, Austria.*

[d]*Dipartimento di Scienze e Tecnologie Biologiche, Chimiche e Farmaceutiche, Viale delle Scienze, Edificio 17, 90128 Palermo, Italy.*

Supporting Info

X-Ray details	pag. 2-9
UV-Vis from CD measurements	pag. 10-11
UV-Vis titrations and K_b	pag. 11-14
Cytotoxicity tests	pag. 15
Docking	pag. 15

Table S1. Experimental parameters and CCDC-Codes

Sample	Machine	Source	Temp.	Detector Distance	Time/ Frame	#Frames	Frame width	CCDC
	Bruker		[K]	[mm]	[s]		[°]	
**L1**	X8	Mo	130	35	60	617	0.5	1451696
**1**	D8	Mo	100	34	96	744	0.4	1451694
**2**	X8	Mo	130	35	30	2289	0.5	1451695

[N,N'-bis(5-triethylammoniummethylsalicylidene)-1,2-ethylenediamine](ClO_4)$_2$ (L1)

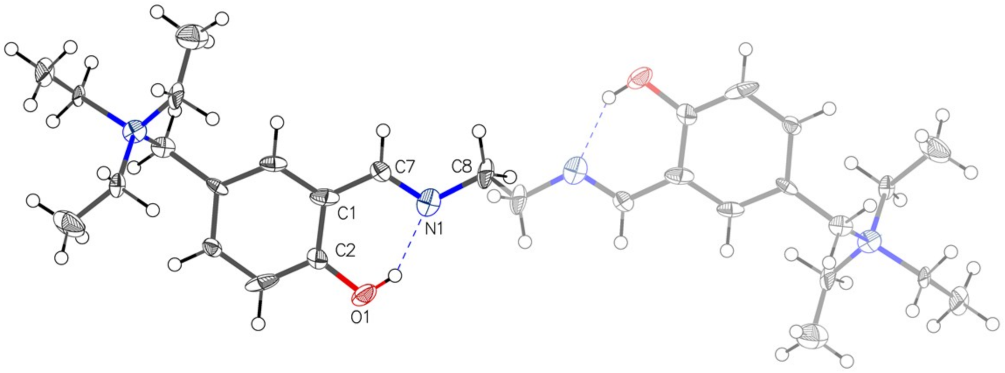

Figure S1. Molecular Structure of **L1**, drawn with 50% displacement ellipsoids. The Asymmetric Unit is highlighted. Perchlorate omitted for clarity. The single bond between C8 and the symmetric equivalent C8` (2-x,-y,1-z) is arranged as "anti" conformer (180° exact, because of symmetry reasons). The solved crystal grew as twin in two domains and the result was solved with the help of Twinabs[1] and cell_now.[2] The twin law transformation after the Saint integration is documented in Table S2. The ratio in the used reflection data, based on the reflections used only in one domain respectively, is 0.99. This indicates a cleavage by 1:1.

Table S2. Twin law transformation after the Saint integration for **L1**

**Domain 1 to Domain 2**		
-1.00015	0.00013	0.00049
-0.00046	-1.00012	0.00048
-0.38653	-0.7621	1.00028

Table S3. Sample and crystal data of **L1**.

**Chemical formula**	C30H48Cl2N4O10	**Crystal system**	triclinic	
**Formula weight [g/mol]**	695.62	**Space group**	*P-1*	
**Temperature [K]**	130	**Z**	1	
**Measurement method**	\Φ and \ω scans	**Volume [Å^3]**	864.2(12)	
**Radiation (Wavelength [Å])**	MoKα (λ = 0.71073)	**Unit cell dimensions [Å] and [°]**	7.779(6)	75.82(2)
**Crystal size / [mm^3]**	0.2 × 0.11 × 0.01		8.339(6)	81.71(2)
**Crystal habit**	clear yellow plate		14.025(12)	80.21(4)
**Density (calculated) / [g/cm^3]**	1.337	**Absorption coefficient / [mm^{-1}]**	0.247	
**Abs. correction Tmin**	0.530345	**Abs. correction Tmax**	0.745987	
**Abs. correction type**	multiscan	**F(000) [e$^-$]**	370	

Table S4. Data collection and structure refinement of **L1**.

**Index ranges**	-9 ≤ h ≤ 9, -9 ≤ k ≤ 9, 0 ≤ l ≤ 16	**Theta range for data collection [°]**	5.088 to 50.7	
**Reflections number**	1942	**Data / restraints / parameters**	1942/30/200	
**Refinement method**	Least squares	**Final R indices**	all data	R1 = 0.1333, wR2 = 0.2268
**Function minimized**	Σ w(F_o^2 - F_c^2)^2		I>2σ(I)	R1 = 0.0928, wR2 = 0.2098
**Goodness-of-fit on F^2**	0.961	**Weighting scheme**	w=1/[σ^2(F_o^2)+(0.1269P)^2]	
**Largest diff. peak and hole [e Å^{-3}]**	1.21/-0.43		where P=(F_o^2+2F_c^2)/3	

[(N,N'-bis(5-triethylammoniummethylsalicylidene)-1,2-ethylenediiminato)nickel(II)] (ClO$_4$)$_2$ **(1)**

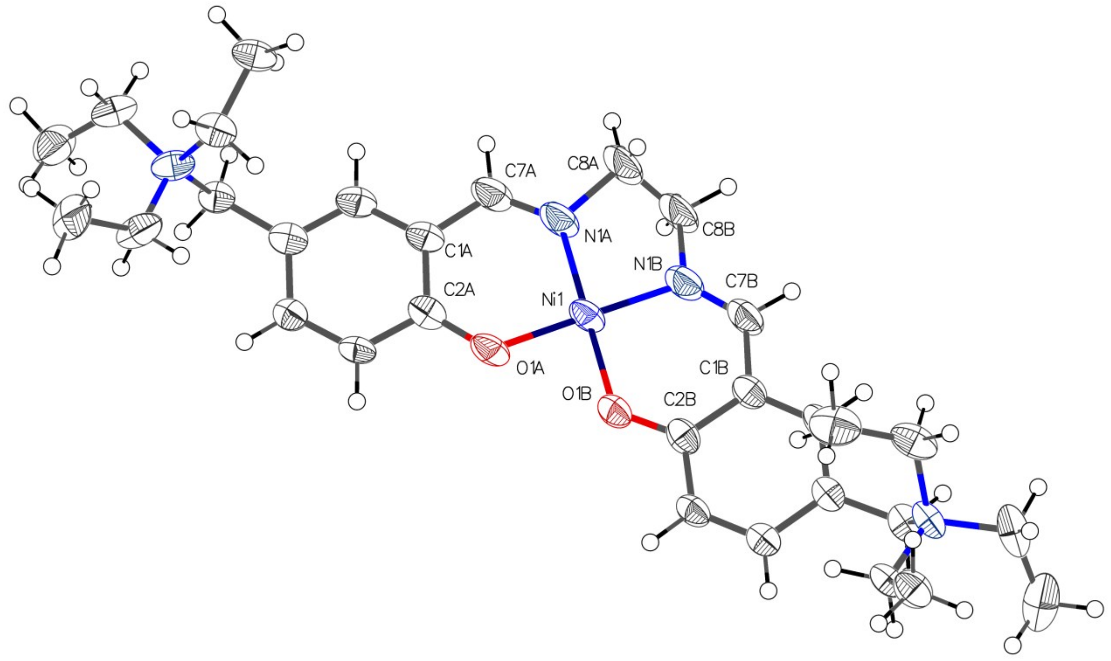

Figure S2. Asymmetric Unit of **1**, drawn with 50% displacement ellipsoids. Perchlorate and disorder omitted for clarity. The single bond between C8A C8B is arranged as "gauche" conformer in the range from 54.4817(18)° for part 1 to 9.5894(6) for part 2).

Table S5. Sample and crystal data of **1**

**Chemical formula**	C30H46Cl2N4NiO10	**Crystal system**	monoclinic	
**Formula weight [g/mol]**	752.32	**Space group**	*P21/n*	
**Temperature [K]**	100	**Z**	4	
**Measurement method**	\Φ and \ω scans	**Volume [Å^3]**	3557.6(3)	
**Radiation (Wavelength [Å])**	MoKα (λ = 0.71073)	**Unit cell dimensions [Å] and [°]**	10.0932(6)	90
**Crystal size / [mm^3]**	0.1 × 0.07 × 0.01		15.4168(8)	92.2906(17)
**Crystal habit**	clear orange plate		22.8812(13)	90
**Density (calculated) / [g/cm^3]**	1.405	**Absorption coefficient / [mm^{-1}]**	0.754	
**Abs. correction Tmin**	0.659	**Abs. correction Tmax**	0.7452	
**Abs. correction type**	multiscan	**F(000) [e$^-$]**	1584	

Table S6. Data collection and structure refinement of **1**

**Index ranges**	$-12 \leq h \leq 12$, $-16 \leq k \leq 18$, $-27 \leq l \leq 27$	**Theta range for data collection [°]**	3.186 to 50.878	
**Reflections number**	28271	**Data / restraints / parameters**	6433/7/453	
**Refinement method**	Least squares	**Final R indices**	all data	R1 = 0.1036, wR2 = 0.1982
**Function minimized**	$\Sigma w(F_o^2 - F_c^2)^2$		I>2σ(I)	R1 = 0.0708, wR2 = 0.1767
**Goodness-of-fit on F^2**	1.072	**Weighting scheme**	$w=1/[\sigma^2(F_o^2)+(0.0766P)^2+11.6114P]$	
**Largest diff. peak and hole [e Å^{-3}]**	1.73/-0.51		where $P=(F_o^2+2F_c^2)/3$	

[(N,N'-bis(5-triethylammoniummethylsalicylidene)-1,2-ethylenediiminato)copper(II)] $(ClO_4)_2$ **(2)**

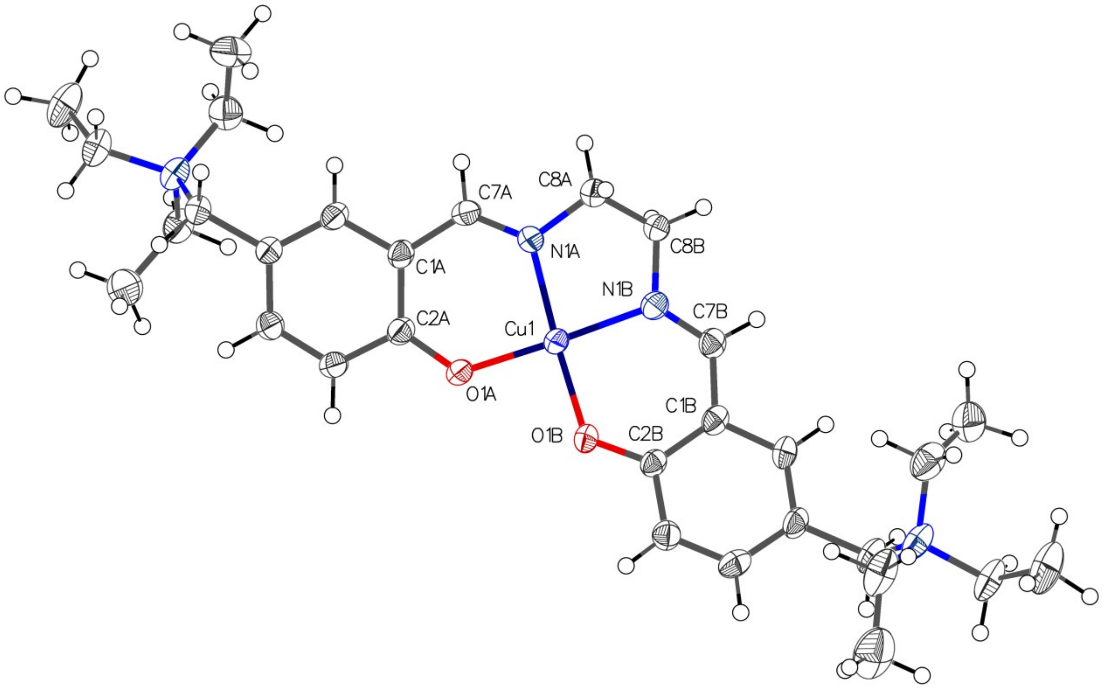

Figure S3. Asymmetric Unit of **2**, drawn with 50% displacement ellipsoids. Perchlorate omitted for clarity. The single bond between C8A C8B is arranged as "gauche" conformer (44.053(7)°). It was necessary to mask (by Olex2) one void because no adequate solution could be found. The volume of this void is 179.6 [Å^3] and the number of excluded electrons is 29.4. Both hkl-Files (original and masked) are uploaded to the CCDC.

Table S7. Sample and crystal data of **2**

**Chemical formula**	C30H46Cl2CuN4O10	**Crystal system**	triclinic	
**Formula weight [g/mol]**	757.15	**Space group**	*P-1*	
**Temperature [K]**	100	**Z**	2	
**Measurement method**	\Φ and \ω scans	**Volume [Å^3]**	1778.6(5)	
**Radiation (Wavelength [Å])**	MoKα (λ = 0.71073)	**Unit cell dimensions [Å] and [°]**	9.2787(13)	70.114(8)
**Crystal size / [mm^3]**	0.35 × 0.13 × 0.03		12.812(2)	82.910(7)
**Crystal habit**	clear brown plate		16.033(3)	87.960(7)
**Density (calculated) / [g/cm^3]**	1.414	**Absorption coefficient / [mm^{-1}]**	0.822	
**Abs. correction Tmin**	0.5589	**Abs. correction Tmax**	0.7254	
**Abs. correction type**	multiscan	**F(000) [e$^-$]**	794	

Table S8. Data collection and structure refinement of **2**

**Index ranges**	$-13 \leq h \leq 13$, $-18 \leq k \leq 18$, $-22 \leq l \leq 22$	**Theta range for data collection [°]**	4.912 to 50.696	
**Reflections number**	41294	**Data / restraints / parameters**	6415/0/430	
**Refinement method**	Least squares	**Final R indices**	all data	R1 = 0.0970, wR2 = 0.2385
**Function minimized**	Σ w(F_o^2 - F_c^2)^2		I>2σ(I)	R1 = 0.0828, wR2 = 0.2265
**Goodness-of-fit on F^2**	1.064	**Weighting scheme**	$w=1/[\sigma^2(F_o^2)+(0.1550P)^2]$	
**Largest diff. peak and hole [e Å^{-3}]**	0.75/-0.68		where $P=(F_o^2+2F_c^2)/3$	

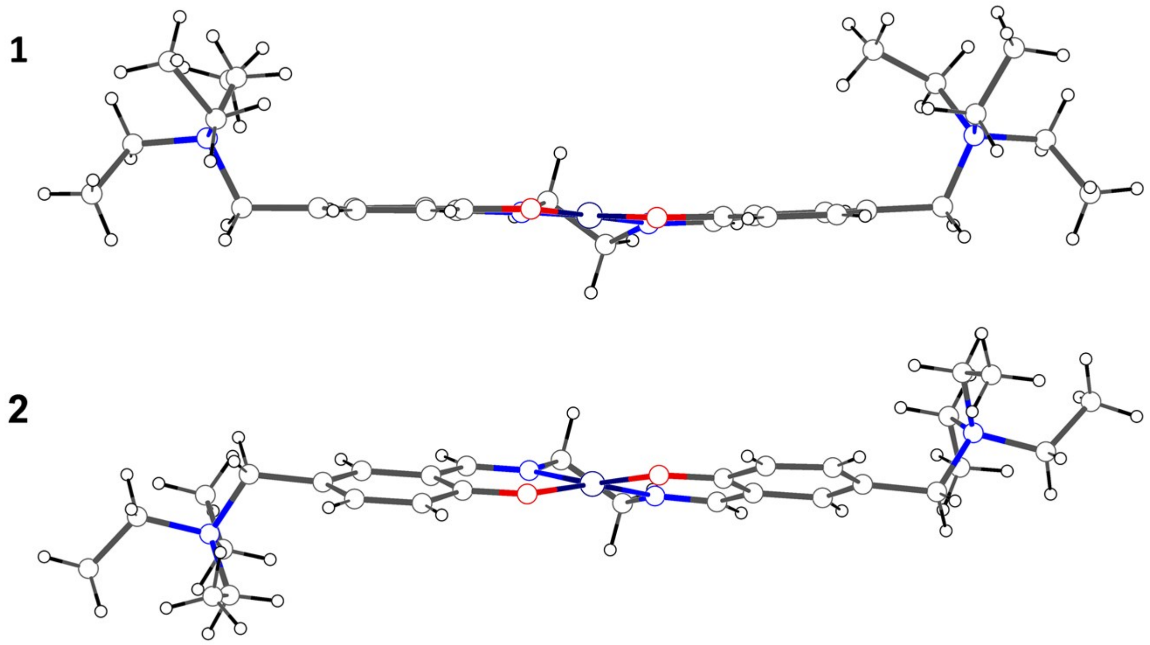

Figure S4. Torsion in the Coordination sphere of **1** (Ni) and **2** (Cu)

Table S9. Torsion in the coordination sphere of **1** and **2**

**Torsion in the coordination sphere of 1 and 2 [°]**		
Ni	N1A-N1B-O1B-O1A	1.07750(6)
Cu	N1A-N1B-O1B-O1A	19.286(4)

Table S10. Ortho atom to mean plane distances for **1** and **2**

**1 (Ni) [Å]**		**2 (Cu) [Å]**	
Ni1	0.03	Cu1	0
N1A	0.011	N1A	0.230
N1B	-0.012	N1B	-0.229
O1A	-0.012	O1A	-0.221
O1B	0.011	O1B	0.221
**Sum Abs. Values**	**0.076**	**Sum Abs. Values**	**0.901**

Table S11. Coordination sphere of 1 and 2 [Å]

Ni(1)	O(1A)	1.835(4)
Ni(1)	O(1B)	1.839(4)
Cu(1)	O(1A)	1.891(3)
Cu(1)	O(1B)	1.906(3)
Ni(1)	N(1A)	1.848(5)
Ni(1)	N(1B)	1.839(5)
Cu(1)	N(1A)	1.940(4)
Cu(1)	N(1B)	1.936(4)

Table S12. Chelate distance

**L1**	N1	O1	2.5823(18)
**1** (Ni)	N(1A)	O(1A)	2.71996(10)
	N(1B)	O(1B)	2.70711(10)
**2** (Cu)	N(1A)	O(1A)	2.8037(4)
	N(1B)	O(1B)	2.8058(4)

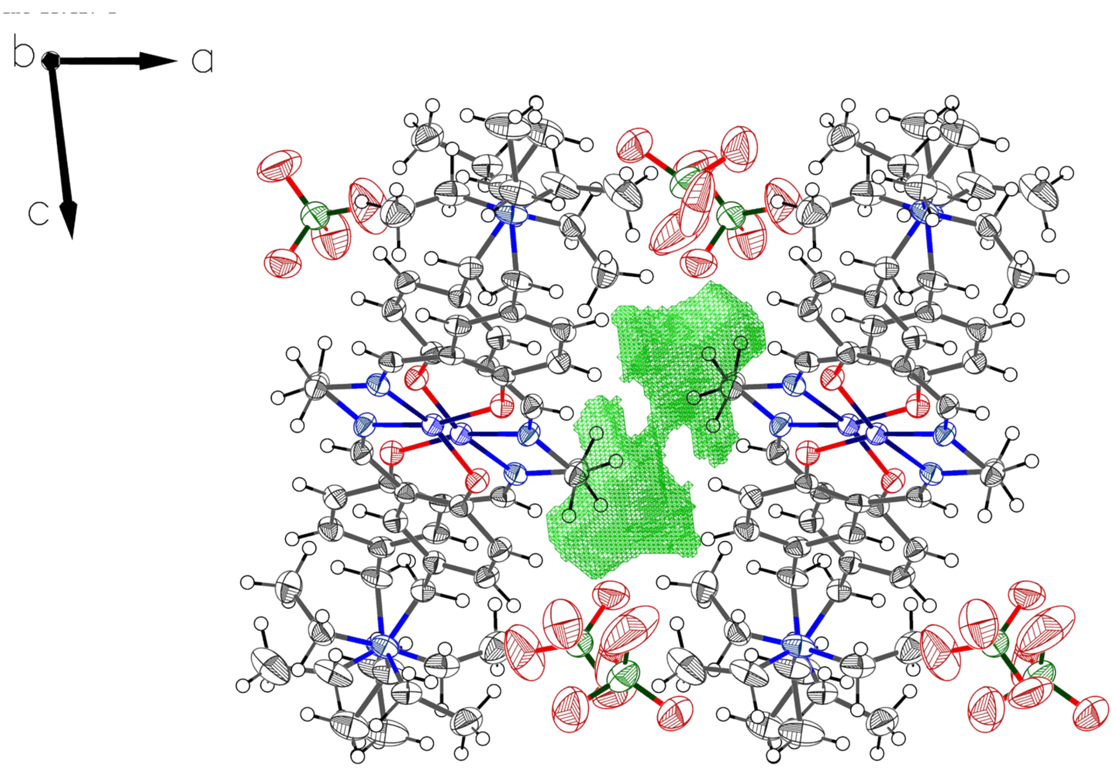

Figure S5. Packing of **2**. The solvent accessible void (green wired volume) is located along axis b. The positions of the free water molecules (used in synthesis and proofed by elemental analysis) could not be fixed.

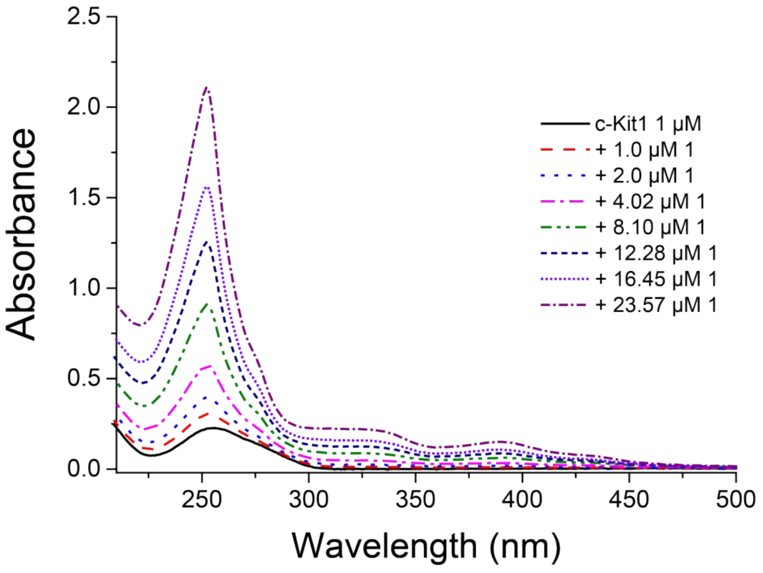

Figure S6. UV-Vis from CD titration of *c-Kit1* with compound **1.**

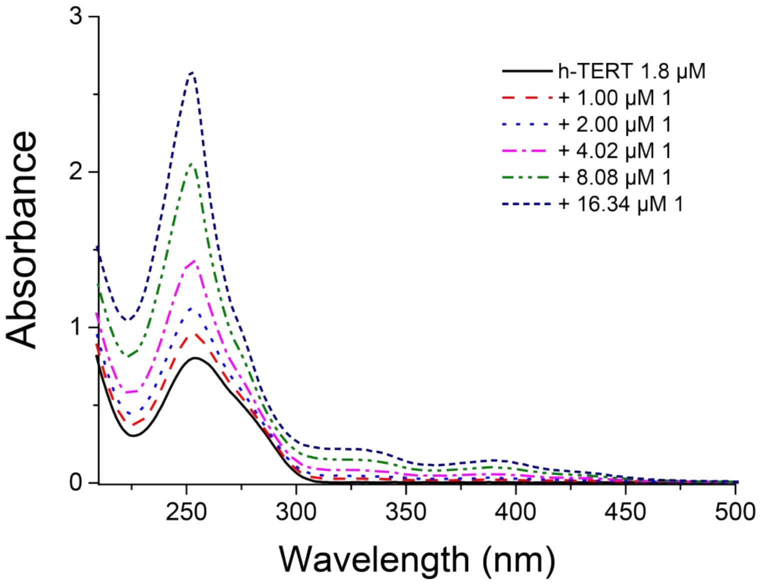

Figure S7. UV-Vis from CD titration of h-*TERT* with compound **1**

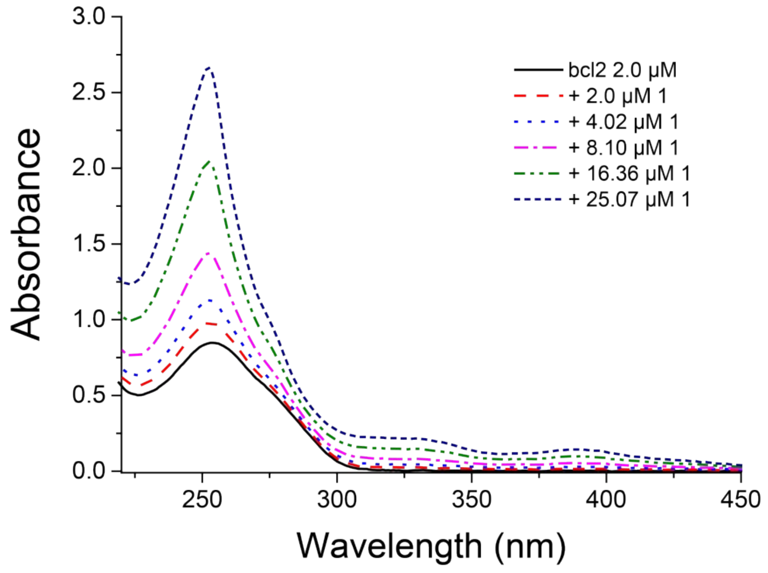

Figure S8. UV-Vis from CD titration of *bcl2* with compound **1**

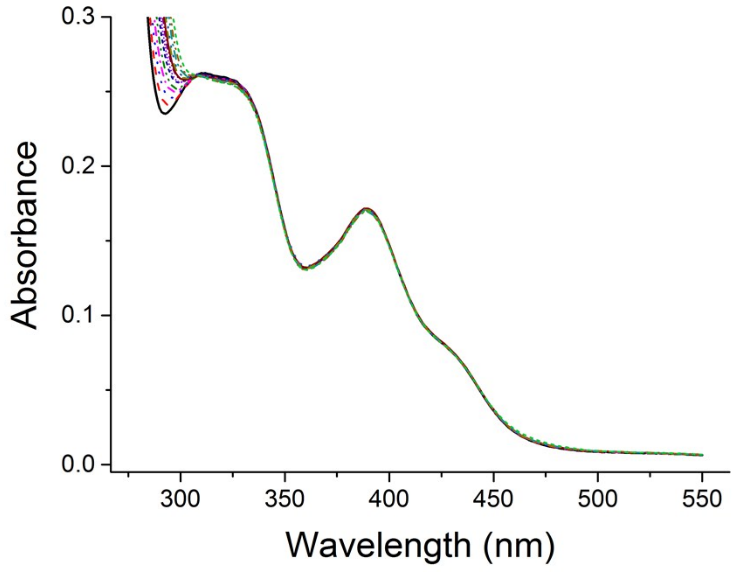

Figure S9. Absorption spectrum of **1** (black solid line) 27.0 μM in presence of increasing amounts of ct-DNA in Tris-HCl buffer 50 mM and KCl 100 mM. DNA concentration is in the range 7.5-90.1 μM

Determination of the binding constant K_b.

Binding constants were obtained using two methods. The first consisted in fitting the data to a reciprocal plot of $[DNA]/|\varepsilon_a-\varepsilon_f|$ vs. [DNA] using the equation:[3]

$[DNA]/|\varepsilon_a-\varepsilon_f| = [DNA]/|\varepsilon_b-\varepsilon_f| + 1/(|\varepsilon_b-\varepsilon_f| \times K_b)$ (Equation 1)

where $\varepsilon_a = A_{observed}/[\mathbf{1}]$, ε_b is the extinction coefficient of the DNA bound complex, and ε_f is the extinction coefficient of the free complex determined by a calibration curve of the isolated metal complexes in aqueous solution, following the Beer–Lambert law.

The second method used is the so-called "Intrinsic method" developed by Rodger and Nordén.[4] The constant is defined by the Equation 2:

$$K_b = \frac{MC_b}{[DNA]_f\, MC_f} \qquad \text{(Equation 2)}$$

where MC_b is the concentration of the bound metal complex, MC_f is the concentration of the free metal complex MC_f and $[DNA]_f$ represents the concentration of free DNA. The relation between MC_b and the change in absorbance (ρ) is $MC_b = \rho\,\alpha$, where α is a constant (for a given wavelength). The relation between the change in DNA concentration and ρ is given by Equation 3. By plotting the x and the y values determined by Equation 4 and 5 respectively, is possible to calculate α and, hence, MC_b.

$$\frac{[DNA]^k_{tot} - [DNA]^j_{tot}}{\rho^k - \rho^j} = \frac{MC_{tot}}{\alpha}\left(\frac{\frac{[DNA]^k_{tot}}{\rho^k} - \frac{[DNA]^j_{tot}}{\rho^j}}{\rho^k - \rho^j}\right) + n\alpha \qquad \text{(Equation 3)}$$

$$x = \left(\frac{\frac{[DNA]^k_{tot}}{\rho^k} - \frac{[DNA]^j_{tot}}{\rho^j}}{\rho^k - \rho^j}\right) \qquad \text{(Equation 4)}$$

$$y = \frac{[DNA]^k_{tot} - [DNA]^j_{tot}}{\rho^k - \rho^j} \qquad \text{(Equation 5)}$$

By a Scatchard plot of r/MC_f versus r, where $r=MC_b/[DNA]_{tot}$, it is possible to estimate the value of the K_b according to the following equation:

$$\frac{r}{MC_f} = \frac{K_b}{n} - rK_b \qquad \text{(Equation 6)}$$

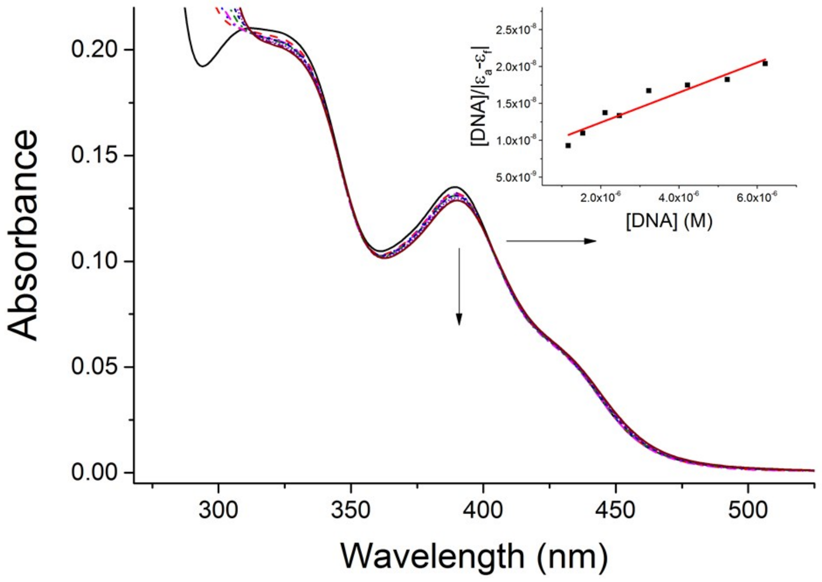

Figure S10. Absorption spectrum of **1** (black solid line) 21.3 µM in presence of increasing amounts of *c-Kit1* in Tris-HCl buffer 50 mM and KCl 100 mM. DNA concentration is in the range 0.37-7.20 µM. The arrows indicate the change upon G4 addition. Inset: plot of the titration data at 389 nm by using Equation 1.

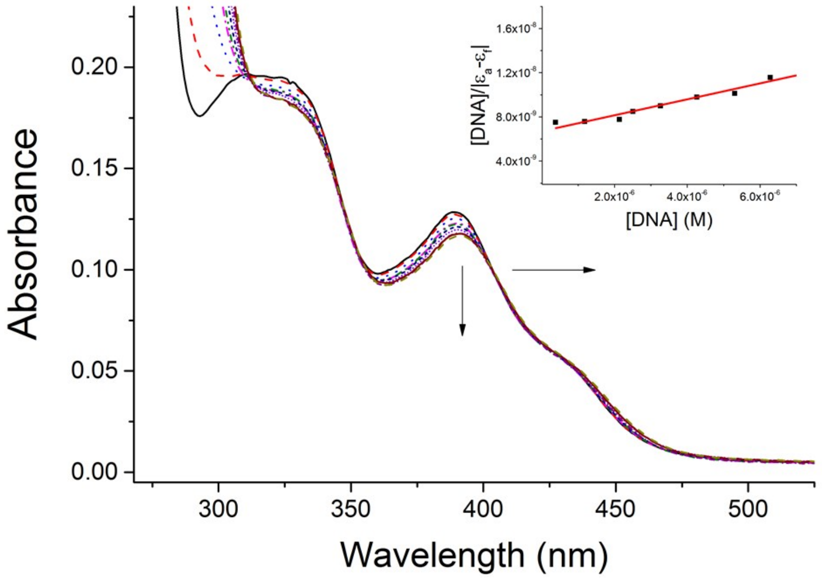

Figure S11. Absorption spectrum of **1** (black solid line) 20.3 µM in presence of increasing amounts of *h-TERT* in Tris-HCl buffer 50 mM and KCl 100 mM. DNA concentration is in the range 0.38-7.29 µM. The arrows indicate the change upon G4 addition. Inset: plot of the titration data at 389 nm by using Equation 1.

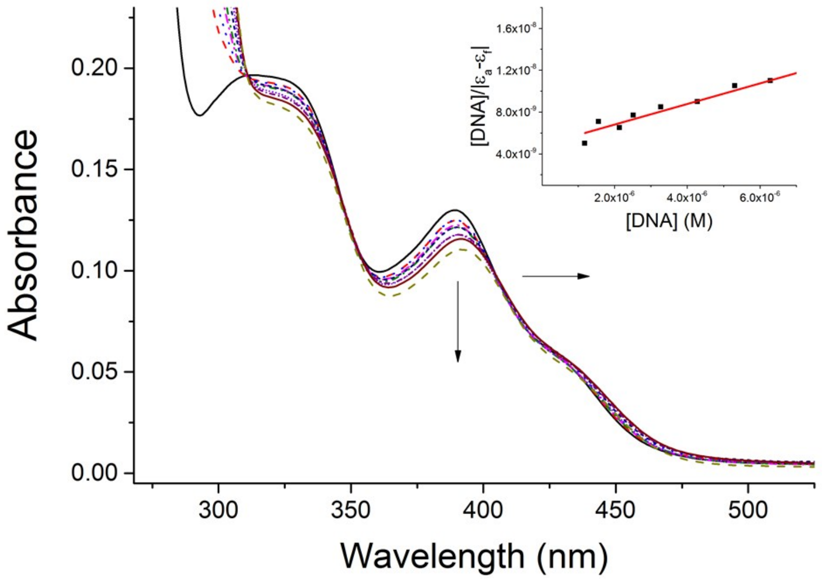

Figure S12. Absorption spectrum of **1** (black solid line) 20.5 µM in presence of increasing amounts of *bcl2* in Tris-HCl buffer 50 mM and KCl 100 mM. DNA concentration is in the range 0.38-7.29 µM. The arrows indicate the change upon G4 addition. Inset: plot of the titration data at 389 nm by using Equation 1.

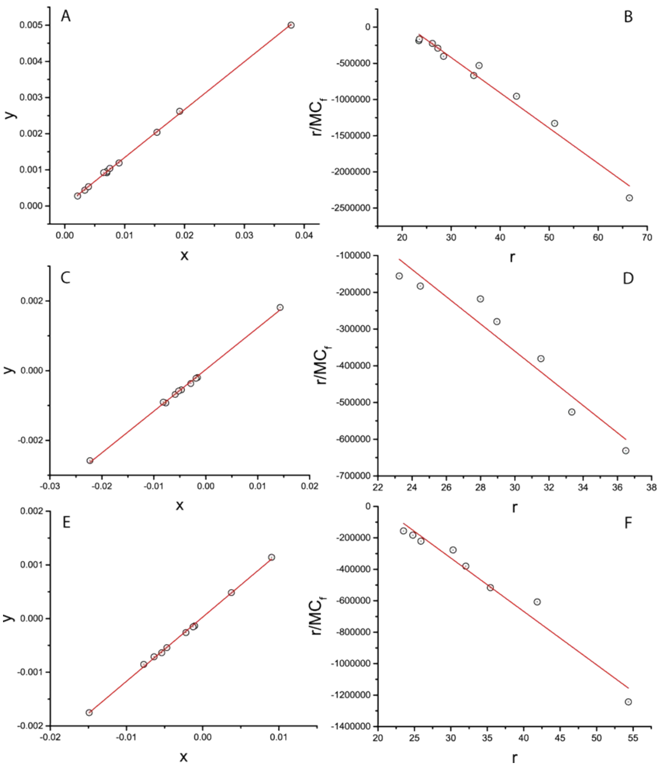

Figure S13. Intrinsic method plots (left column) of x and y as defined in Equations 4 and 5, and Scatchard plots (right column) according to Equation 6 for **1** with increasing amounts of c-Kit1 (A, B), h-TERT (C, D) and bcl2 (E, F) quadruplexes. α values were $(1.61 \pm 0.9) \times 10^{-4}$ for c-Kit1, $(1.69 \pm 0.9) \times 10^{-4}$ for h-TERT and $(1.71 \pm 0.6) \times 10^{-4}$ for bcl2.

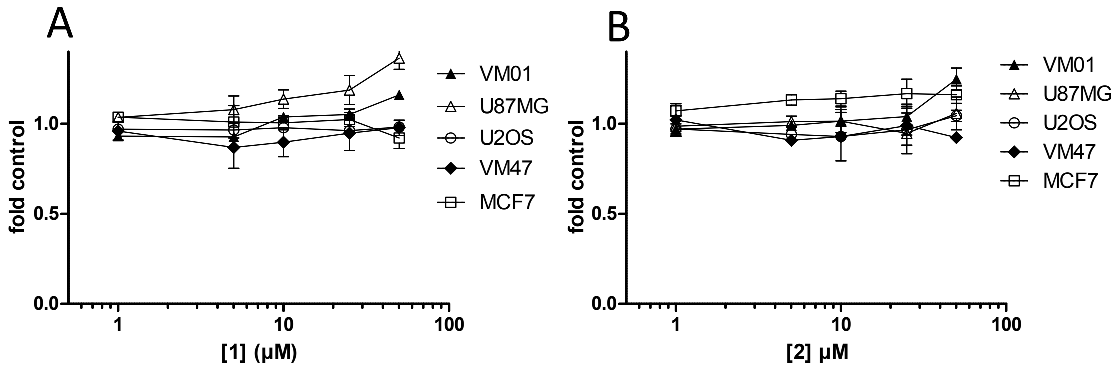

Figure S14. Cytotoxic activity of compound **1** (A) and **2** (B) upon 72 hours exposure. VM01 and VM47 are melanoma cell lines. U87MG is a glioblastoma, U2OS an osteosarcoma and MCF7 a breast cancer cell line.

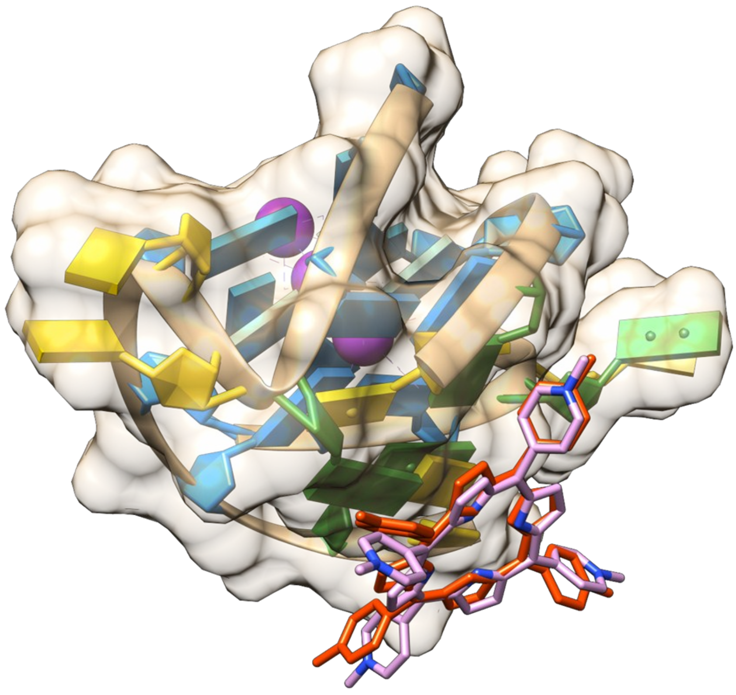

Figure S15. Cartoons showing docking pose of compound TMPyP4 (violet) in complex with the parallel stranded human telomeric quadruplex (PDB ID: 2HRI). The ligand in the original PDB is represented in orange. Bases Colours: G = blue, T = gold, A = green.

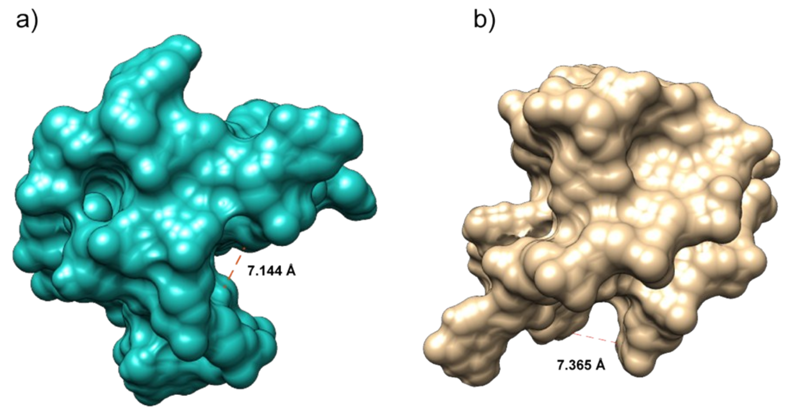

Figure S16. Cartoons showing grooves width of a) *c-Kit1* (PDB entry 2O3M) and b) the bcl2 (PDB entry 2F8U) G4 motifs, both represented with van der Waals surface.

References

[1] Sheldrick, G. M. (1996). SADABS, TWINABS. University of Göttingen, Germany.

[2] Sheldrick, G. M. (2008). CELL_NOW. Version 2008/4. Universität Göttingen, Germany.

[3] Wolfe, G. H. Shimer and T. Meehan, Biochemistry, 1987, 26, 6392-6396.

[4] Rodger, A.; Nordén, B., Circular and Linear Dichroism. Oxford University Press: Oxford: 1997; 135-140.
